# Repeated Exposure to Optic Flow in Virtual Reality Induces Changes in Postural Control in Older Adults

**DOI:** 10.3390/s26123772

**Published:** 2026-06-12

**Authors:** Christophe Barbanchon, Stéphane Baudry

**Affiliations:** NeuroMove—Laboratory of Movement Neurophysiology, Faculty of human Movement Sciences, Université Libre de Bruxelles, 1070 Bruxelles, Belgium; christophe.barbanchon@ulb.be

**Keywords:** sensory reweighting, ageing, balance, proprioception

## Abstract

**Highlights:**

**What are the main findings?**

- Repeated exposure to optic flow in an immersive virtual environment attenuates visually induced postural destabilization during simulated forward and backward self-motion in healthy older adults.
- The intervention selectively improves quiet-standing stability when vision is removed and reorganizes sensory strategy by enhancing visual intra-modal consistency while suppressing a pre-existing visual–proprioceptive coupling.

**What are the implications of the main findings?**

- These results demonstrate that sensory reweighting mechanisms and balance strategies retain short-term plasticity in older age when repeatedly challenged by visual perturbations.
- Virtual-reality-based optic flow exposure emerges as a promising and targeted approach to promote more flexible balance control strategies in aging populations.

**Abstract:**

This study investigates the effect of repeated exposure to optic flow in virtual reality (VR) on postural control in upright standing in older adults. Eighteen participants (>60 years) completed pre/post assessments consisting of quiet standing in a real environment (eyes open/closed), postural responses to simulated forward and backward self-motion in VR, and proprioceptive perturbations induced by bilateral Achilles and tibialis anterior tendon vibration. Intra- (within similar sensory modality) and inter-modal correlations (within different sensory modalities inducing similar directional postural response) were also investigated to provide insight into sensory integration strategies. The intervention consisted of six 100-s VR bouts alternating simulated forward and backward self-motion. Postural outcomes were quantified from force platform recordings as center of pressure (CoP) velocity and excursion. Repeated VR exposure reduced CoP velocity during simulated forward and backward self-motion (*p* < 0.05). After the intervention, CoP velocity decreased when standing with eyes closed (*p* < 0.05) but not when eyes were open. Postural response to tendon vibration was not modified by the intervention (*p* > 0.05). After the intervention, intra-modal correlation for postural responses to optic flow appeared, whereas a pre-existing inter-modal association between Achilles vibration and forward optic flow disappeared. These results indicate that the postural control system remains adaptable in older adults and highlight the potential of VR environments to improve balance in older adults.

## 1. Introduction

Standing balance depends on the continuous and complex integration of visual, vestibular, and somatosensory (proprioceptive and cutaneous) information to estimate body orientation and generate corrective torques, a framework formalized by Peterka in their influential sensorimotor control model of upright stance [1]. In a few words, the nervous system does not simply sum sensory cues but rather assigns weights to each sensory channel according to their relative reliability and the demands of the task [2,3,4,5]. In agreement, Fransson et al. showed a reduction in postural instability following repeated exposures to a virtual-reality rollercoaster, indicating adaptive changes in balance control [6]. A more recent study [7] reported that six 90-s bouts of optic flow simulating forward and backward self-motion in a virtual environment reduced postural response to optic flow. In addition, the intervention decreased the Romberg ratio (reduced center of pressure instability when standing with eyes closed relative to eyes open) during quiet standing in a real environment in young adults. These findings highlighted the potential of virtual reality as a tool to modify the dynamic of the sensory reweighting during upright standing.

With advancing age, postural control deteriorates and the risk of falling increases, partly due to age-related declines in the efficiency and speed of multisensory integration [8]. Among these changes, older adults tend to depend more heavily on visual information to maintain postural stability than younger individuals [9,10], although this finding has not been consistently confirmed across studies [11,12]. Using a virtual environment to generate optic flow in upright standing, an exaggerated postural response was reported in older compared with young adults during simulated forward self-motion [13]. Even though a comparable age effect during backward simulated self-motion was not observed, which may be explained by a less ecological condition [14] and a greater fear of falling backward in older adults [15], this result supports an age-related shift in sensory weighting favoring vision [9,16]. Such greater reliance on vision could contribute to decreasing their sensory reweighting flexibility [8]. In this context, the use of repeated exposure to optic flow could be relevant for older adults. However, the assumed greater reliance on visual inputs to control upright standing in older adults may reduce this adaptive response to repeated visual threat.

A common assumption underlying the age-related increase in visual reliance for balance is a reduced contribution of proprioceptive inputs. This can be assessed with tendon vibration [17], with the assumption that tendon vibration should have less effect on postural control in older adults [10,18]. Contrary to this view, we observed no age-related differences in postural responses to vibration [13], in contrast to previous work [19,20]. Nonetheless, tendon vibration robustly activates Ia muscle spindle afferents, reliably eliciting kinaesthetic illusions and postural adjustments [21,22], which may override subtle age-related changes in central integration. These results, however, do not preclude an adaptive reweighting of proprioceptive information in response to repeated simulated forward and backward self-motion. Because older adults show greater visual dependence during standing balance than younger adults [9], repeated exposure to visually provocative simulated self-motion may progressively reduce the destabilizing influence of vision through habituation and sensory reweighting. Within the sensory reweighting framework, down-weighting a less reliable sensory channel increases the relative contribution of the remaining channels to stabilize posture. In this context, repeated optic flow exposure could therefore shift older adults toward greater reliance on non-visual cues, including proprioceptive inputs [1]. Such a shift would increase the magnitude of the postural response to tendon vibrations applied to muscles surrounding the ankle.

Furthermore, concurrently assessing changes in postural responses to visual and proprioceptive perturbations after repeated exposure to virtual optic flow could help to determine intra-modal relations (e.g., Achilles vs. tibialis anterior vibration; forward vs. backward optic flow) and inter-modal relations (visual vs. proprioceptive perturbations) [23,24]. Intra-modal relationships capture the consistency of processing within a single sensory system, as each modality relies on its own internal model [1]. By contrast, inter-modal relationships reflect adjustments made by the CNS, typically in opposite directions, in the relative weighting of different sensory inputs. Previous studies indicate that proprioceptive and visual reweighting tend to covary strongly within modalities, whereas interactions between modalities are often weak, asymmetric, or only evident under conditions of explicit conflict. This pattern underscores the functional distinction between intra- and inter-modal relationships [23,24]. We previously reported an altered intra-modal consistency and the emergence of positive inter-modal relations in older adults, which could reflect a reduced modality-specific stability and diminished flexibility in integrating visual and proprioceptive information [13]. Whether these age-related changes can be modified by repeated exposure to visual threat remains to be determined.

Accordingly, the present study examined whether an acute intervention consisting of six bouts of optic flow exposure in an immersive virtual environment modifies postural control in healthy older adults. More specifically, it was tested whether such an intervention modified (1) postural responses to simulated forward and backward self-motion in VR, (2) quiet-standing stability in a real environment during eyes open (EO) and eyes closed (EC) conditions, and (3) responses to proprioceptive perturbations induced by Achilles and TA tendon vibration. In addition, it was evaluated whether it changes the intra-modal and inter-modal associations used as indices of sensory strategy in older adults.

## 2. Material and Methods

### 2.1. Sample

A total of 18 participants aged over 60 years (66.3 (5.5) years, 11 women) volunteered to take part in this study after giving their written informed consent. The sample size was defined a priori based on [7] for a statistical power of 0.8 and an α level of 0.05. Participants were enrolled in the study if they did not have history of falls (absence of fall in the last 12 months preceding the participation to the study), neurological disorders with potential residual motor deficits (stroke, Parkinson’s disease, multiple sclerosis, etc.), diabetes, epilepsy, cardiac history, orthopedic issues involving the lower limbs, and did not take medications that could influence balance (sedatives, hypnotics, antidepressants, and benzodiazepines). All participants were naïve to immersive virtual reality, and none exhibited signs of cybersickness. Approval of the project was obtained from the local Ethics Committee (ULB Erasme committee, CCB B4062024000413), and all procedures used in this study conformed to the Declaration of Helsinki.

### 2.2. Force Platform

A force platform (OR6-6-2000, Advanced Mechanical Technology, Watertown, MA, USA) was used to compute posturography variables. The signals from the platform were sampled at 100 Hz, A/D converted (Power 1401, 16-bit resolution, Cambridge Electronic Design, Cambridge, UK), and stored on a computer to compute offline the position of the center of pressure (CoP).

### 2.3. Virtual Reality

The “Optic Flow” application from the “Balance VR pack” (Virtualis; virtualisvr.com) was employed to immerse participants in a tunnel-like virtual scene featuring longitudinal furrows and no visible endpoint. Forward linear self-motion was generated using centrifugal optic flow, whereas backward linear self-motion was produced using centripetal optic flow. Both motion directions were delivered at 8 m/s, with 2-s acceleration and deceleration phases. The scene was presented via a Vive-Pro head-mounted display (HTC corporation, Taiwan) equipped with a G-sensor (2880 × 2800 total resolution; 1440 × 1600 per eye; AMOLED).

### 2.4. Surface Electromyogram

A Delsys Trigno wireless instrument (Delsys Inc., Natick, MA, USA) was used for recording surface electromyography (EMG) from SOL, gastrocnemius medialis (GM), and tibialis anterior (TA) muscles from the non-dominant leg. The dominant leg was determined by asking an individual to kick a ball [25]. Before attaching the sensors, the skin was shaved when necessary and cleaned with a solution of alcohol, ether, and acetone to reduce the impedance at the skin–electrode interface. The sensor for SOL was placed 2 cm below the muscle–tendon junction of the GM in line with the Achilles tendon. The sensor for the GM was placed midway between the femoral condyle and the muscle–tendon junction. The sensor for the TA was placed one-third of the distance between the fibular head and the lateral malleolus, and 1 cm lateral to the tibia. Surface EMG signals were recorded at a sampling rate of 1926 Hz. The sensor features an analog bandpass (20–450 Hz) with 2nd-order high-pass and 4th-order low-pass filters. The wireless multi-channel EMG signal was A/D converted (Power 1401, 16-bit resolution, Cambridge Electronic Design, UK) before being stored on a computer for subsequent analyses.

### 2.5. Maximal Voluntary Contraction

A custom-made ergometric device was used to measure the torque developed during maximal voluntary isometric contractions (MVC) performed with the ankle plantar flexor muscles or the ankle dorsiflexors of the non-dominant leg. Participants were seated with the ankle and knee angles positioned at 90° and 0° (full knee extension), respectively, and the foot tightly attached to a footplate that was connected to a force transducer (model 4576A2N, Kistler, Winterthur, Switzerland). The force transducer signal was amplified (×100) and low-pass filtered at 200 Hz. For each MVC, participants were asked to contract as hard as possible the plantar flexor muscles or the dorsiflexor muscles for 5 s. A minimum of three MVCs was performed. If the peak torque (see below) of the two greatest MVCs differed by more than 5%, additional trials were performed until the 5% criterion was achieved (all subjects reached the 5% criterion within 5 MVCs). The MVC with the greater peak force was thereafter used for analysis.

### 2.6. Achilles Tendon and Anterior Tibial Tendon Vibration

Custom-made mechanical vibrators were fastened bilaterally on either the Achilles tendons or the anterior tibialis tendons. Vibration at 80 Hz frequency with a 1 mm amplitude was used, as these parameters were shown to be optimal to induce kinesthetic [26] and postural perturbations [17,27]. All vibration tests were conducted with the participants’ eyes closed to prevent visual cues from influencing the postural response.

### 2.7. Experimental Protocol

**Figure 1:**
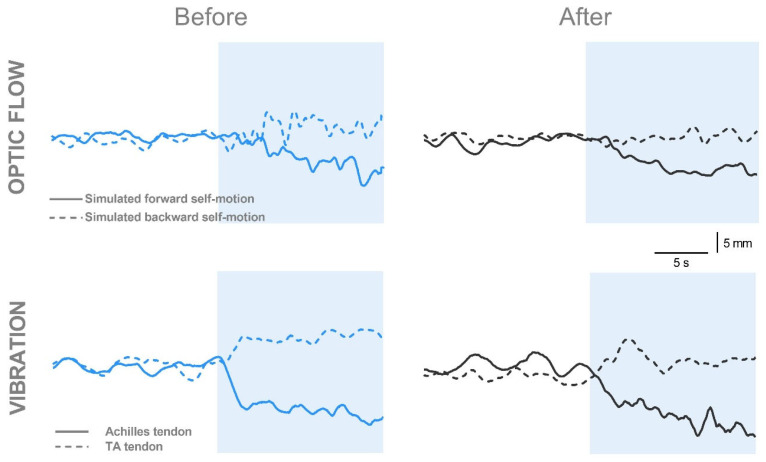
**Center of pressure signal before and after the intervention.** Original traces, from one participant, of the center of pressure in the anterior–posterior direction during the first 30 s of simulated forward and backward self-motion (**top**) panels) and tendon vibration ((**bottom**) panel,) before (**left**) panels) and after (**right**) panels) the six bouts of optic flow in virtual reality. The first 15 s correspond to the period without sensory stimulus, followed by 15 s with sensory stimulus (shaded zone).

The overall protocol was similar to that of Barbanchon and Baudry [7], comprising an acute intervention involving six bouts of simulated forward–backward displacement (optic flow) in the VR environment. Before and after the repeated exposure to optic flow, assessments were conducted for postural response to optic flow in the environment (simulated forward or backward self-motion) (Figure 1), to Achillean vibration, to anterior tibial tendon vibration, and balance performance in eyes open (EO) and eyes closed (EC) conditions in a real environment. Across all experimental conditions, participants stood upright in a bipedal stance with their heels separated by 10 cm and their forefeet turned outward to form a 30° angle between the feet (i.e., each foot externally rotated by 15° relative to the forward direction). They kept their arms relaxed alongside the body and were asked to avoid any head, arm, or leg movements.

*Before the intervention*: Participants were instructed to maintain upright standing as steadily as possible with EO and EC for 60 s each. A single trial for each visual condition was performed in a random order across participants, with a minimum of 30 s of rest between trials.

In random order, simulated forward or backward self-motion or bilateral Achilles or TA tendon vibration was applied, with two series for each type of sensory stimulus. For simulated self-motion, there was a random order of forward or backward direction. Similarly, the bilateral vibrations to the Achilles tendon or anterior tibial tendon were applied in a random order. Regardless of the type of sensory stimulus, each series was divided into three epochs: the first 15 s prior to the sensory stimulus, 15 s during which the sensory stimulus was on, either a simulated forward or backward movement or bilateral vibration at the Achilles tendon or anterior tibial tendon, followed by 30 s without sensory stimulus, allowing participants to regain their balance.

*Repeated exposure to optic flow*: Subsequently, participants underwent six bouts of 100 s in the VR environment. Each bout alternated between forward and backward displacements, with the number of shifts in direction being either 3, 4, or 9. The same direction was maintained for either 10, 20, or 30 s. The same six bouts were used for all participants, but in a randomized order across participants.

*After the intervention*: After the repeated optic flow exposure, participants underwent the same assessments as in the pre-test.

### 2.8. Data Reduction

The displacement of the CoP was analyzed offline using Spike2 software, version 8.01 (Cambridge Electronic Design, Cambridge, UK). Command lines were specifically written to process platform signals into CoP parameters. Initially, force platform signals underwent low-pass filtering (cut-off frequency: 10 Hz) using a fourth-order Butterworth filter. Subsequently, from the filtered data, the CoP mean velocity (CoP velocity) and the maximal amplitude of CoP excursion in AP and ML direction were measured. During upright standing with eyes open and closed, these variables were measured over 40 s, avoiding the first 10 s of each trial. Similarly, the average rectified value of the EMG signal for SOL, GM, and TA was calculated for the same 40-s epoch in EO and EC conditions as those of the CoP calculation and expressed as a percentage of the EMG values of the respective muscles recorded during maximal voluntary contraction. The EMG value during MVC was measured during a 1-s epoch during the peak force [28].

During simulated displacements in VR and tendon vibration, the CoP mean velocity was computed for the 15-s epoch prior to the perturbation and the 15-s epoch of the perturbation, independently for the antero-posterior direction and the medio-lateral direction, and with both directions pooled. The effect of the repeated exposure to optic flow on the response to sensory stimulus was assessed as the extent of the change in CoP mean velocity (ΔCoP_VEL_) in response to the sensory stimulus relative to the value recorded within the 15 s that preceded the stimulus, with the following formula:

$$∆CoPvel\;(\%\;baseline)\;=\frac{CoP\;during\;stimulus-CoP\;before\;stimulus}{CoP\;before\;stimulus}\;\times 100$$

The maximal excursion of CoP in the antero-posterior and medio-lateral directions during the perturbation was calculated as the distance between the baseline CoP position (mean values of the 15 s preceding the sensory stimulus) and the peak value during the 15 s of the stimulus presentation (ΔCoP_AMP_). The EMG during the balance perturbation was not measured due to the artifact generated by the vibrations.

### 2.9. Statistical Analysis

A Shapiro–Wilk test was performed on each dataset to assess the Gaussian distribution. As the distribution of the data was not distributed following a Gaussian function, the effect of repeated exposure to optic flow (before vs. after the intervention) was assessed for each variable defined above with a Wilcoxon signed-rank test. In addition, to provide further insights on the effect of the perturbation, Spearman correlations were performed to assess intra- and inter-modal sensory strategies using ΔCoP_VEL_ in AP direction [13]. Rank-biserial correlation (r_rb_) was used for the main results as a non-parametric effect size, with the sign indicating the direction of the effect and the absolute value indicating its magnitude, with 0.10, 0.30, and 0.50 indicating small, medium, and large effects, respectively. The level of significance was set at *p* ≤ 0.05. Data are expressed as median (interquartile range) in the text and tables.

## 3. Results

### 3.1. Postural Controm with Eyes Open and Eyes Closed

There was no significant change in CoP amplitude with eyes open and closed after compared with before the six bouts of optic flow, regardless of the direction (antero-posterior and medio-lateral; Table 1).

**Figure 2:**
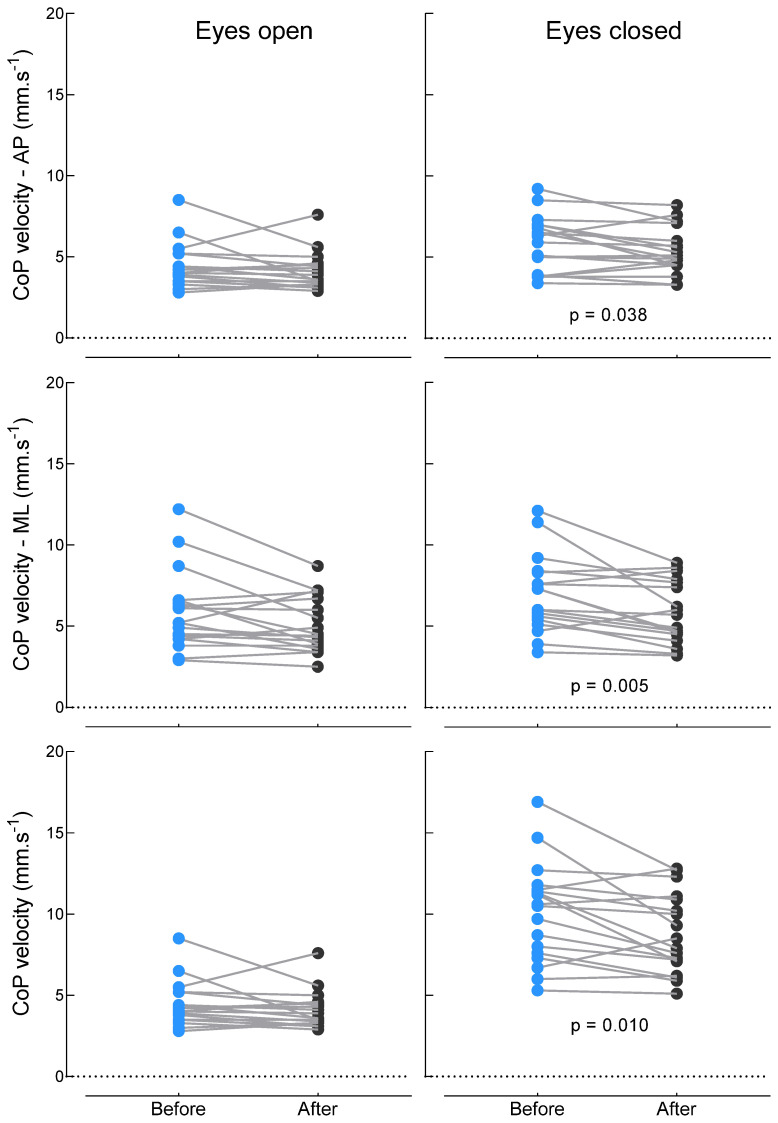
**Center of pressure velocity during upright standing before and after the intervention.** Mean center of pressure velocity (CoP velocity) in antero-posterior (AP; (**top**) panels), medio-lateral (ML; (**mid**) panels) and both directions pooled ((**bottom**) panels), before (blue symbols) and after (dark symbols) the six bouts of optic flow in virtual reality, when standing with eyes open ((**left**) column) and closed ((**right**) column). The *p*-values denote significant differences between before and after the intervention. Each point represents individual data.

Similarly, the CoP velocity was unchanged after the intervention when assessed in the eyes-open condition (Figure 2, left panels). In contrast, in the eyes-closed condition, the CoP velocity decreased (*p* < 0.05, r_rb_ < 0.55) after the intervention in AP and ML directions and for both directions combined (Figure 2, right panels). The Romberg ratio changed neither in AP [before: 1.3 (0.5); after: 1.3 (0.2); *p* = 1.000, r_rb_ = −0.01] nor ML direction [before: 1.2 (0.3); after: 1.1 (0.3); *p* = 0.265, r_rb_ − 0.31], nor for both directions combined [before: 1.2 (0.3); after: 1.2 (0.2); *p* = 0.369, r_rb_ − 0.25].

SOL EMG did not change after the intervention (*p* > 0.05, r_rb_ < 0.18) while the GM EMG decreased in the eyes closed (*p* = 0.028, r_rb_ = 0.56) but not in the eyes open condition (*p* = 0.086, r_rb_ = 0.43). No significant difference was observed after the intervention for TA EMG (*p* > 0.05, r_rb_ < 0.25).

### 3.2. Postural Response to Simulated SELF-Motion

In response to simulated forward self-motion, ΔCoP_AMP_ decreased after the intervention in AP (*p* = 0.005, r_rb_ = 0.78) and ML direction (*p* = 0.017, r_rb_ = 0.70) (Table 2). Similarly, ΔCoP_AMP_ decreased after the intervention in AP (*p* = 0.036, r_rb_ = 0.61) but not ML direction in response to simulated backward self-motion (*p* = 0.231, r_rb_ = 0.21) (Table 2).

**Figure 3:**
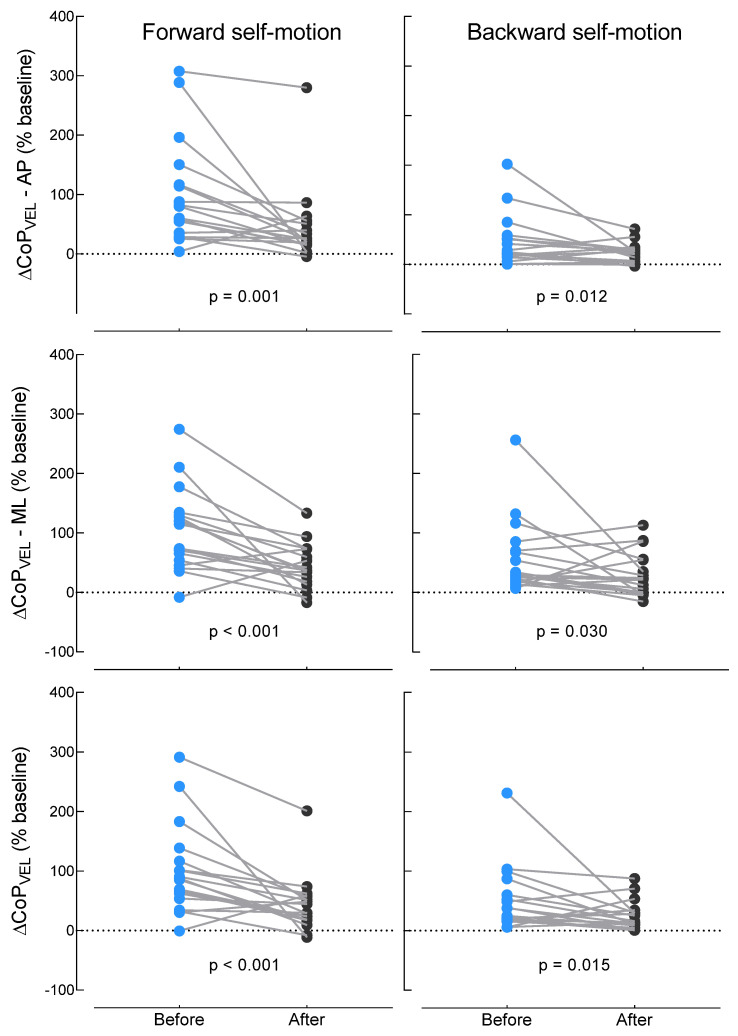
**Effect of simulated self-motion on center of pressure velocity before and after the intervention.** Effect of the simulated displacement (forward: (**left**) column; backward: (**right**) column), expressed as percent of the baseline, on the mean center of pressure velocity (ΔCoP_VEL_) in antero-posterior (AP; (**top**) panels), medio-lateral (ML; (**mid**) panels), and both directions pooled ((**bottom**) panels), before (blue symbols) and after (dark symbols) the the six bouts of optic flow in virtual reality. The *p*-values denote significant differences between before and after the intervention. Each point represents individual data.

After the intervention, ΔCoP_VEL_ decreased in response to simulated forward self-motion in AP (*p* = 0.001, r_rb_ = 0.83), ML (*p* < 0.001, r_rb_ = 0.85), and combined directions (*p* < 0.001, r_rb_ = 0.84). Similarly, ΔCoP_VEL_ decreased after the intervention in response to simulated backward self-motion in AP (*p* = 0.012, r_rb_ = 0.60), ML (*p* = 0.030, r_rb_ = 0.51), and combined directions (*p* = 0.015, r_rb_ = 0.58) (Figure 3).

### 3.3. Postural Response to Tendon Vibration

**Figure 4:**
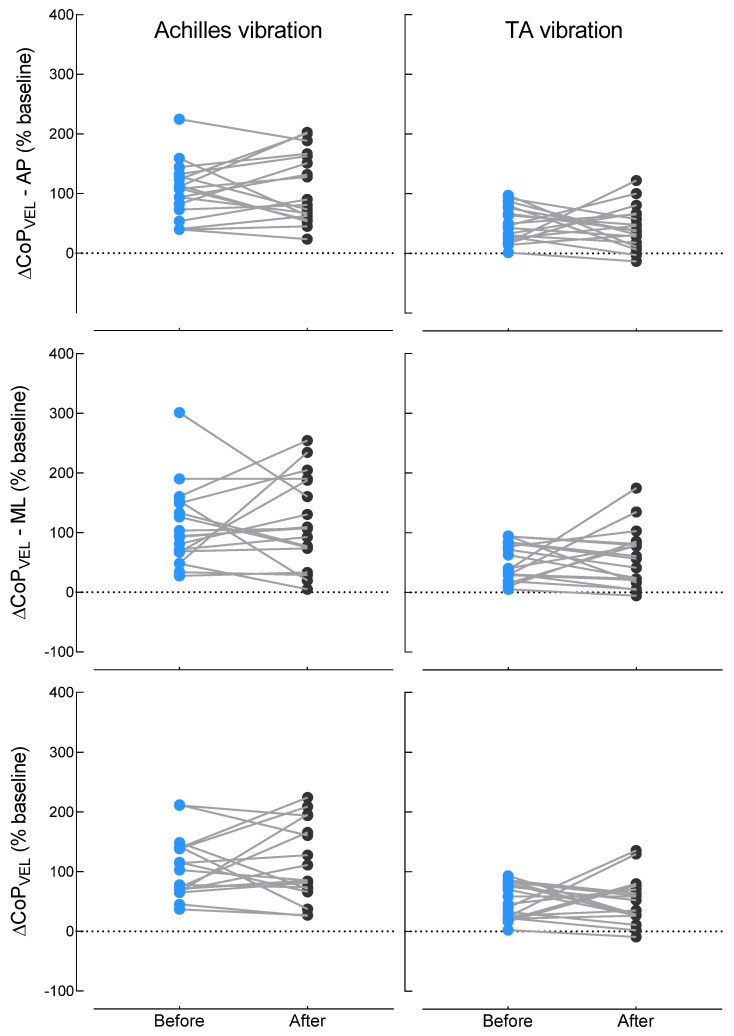
**Effect of tendon vibration on center of pressure velocity before and after the intervention.** Effect of the Achilles ((**left**) column) and tibialis anterior (TA) tendon vibration ((**right**) column), expressed as percent of the baseline, on the mean center of pressure velocity (ΔCoP_VEL_) in antero-posterior (AP; (**top**) panels), medio-lateral (ML; (**mid**) panels), and both directions pooled ((**bottom**) panels), before (blue symbols) and after (dark symbols) the six bouts of optic flow in virtual reality. Each point represents individual data.

In response to Achilles tendon vibration, the ΔCoP_AMP_ did not change after the intervention, regardless of direction (*p* > 0.05, r_rb_~−0.21). Similarly, ΔCoP_AMP_ was not modified in response to TA tendon vibration in AP (*p* = 0.335, r_rb_ = 0.10), ML (*p* = 0.517, r_rb_ = −0.38), and combined direction (*p* = 0.483, r_rb_ = 0.15) (Table 2). After the intervention, ΔCoP_VEL_ in response to Achilles tendon vibration did not change, regardless of the direction (*p* > 0.05, r_rb_~0.15). Similarly, ΔCoP_VEL_ was not different after compared to before the intervention in response to TA tendon vibration (Figure 4).

### 3.4. Correlations Between Postural Responses

**Figure 5:**
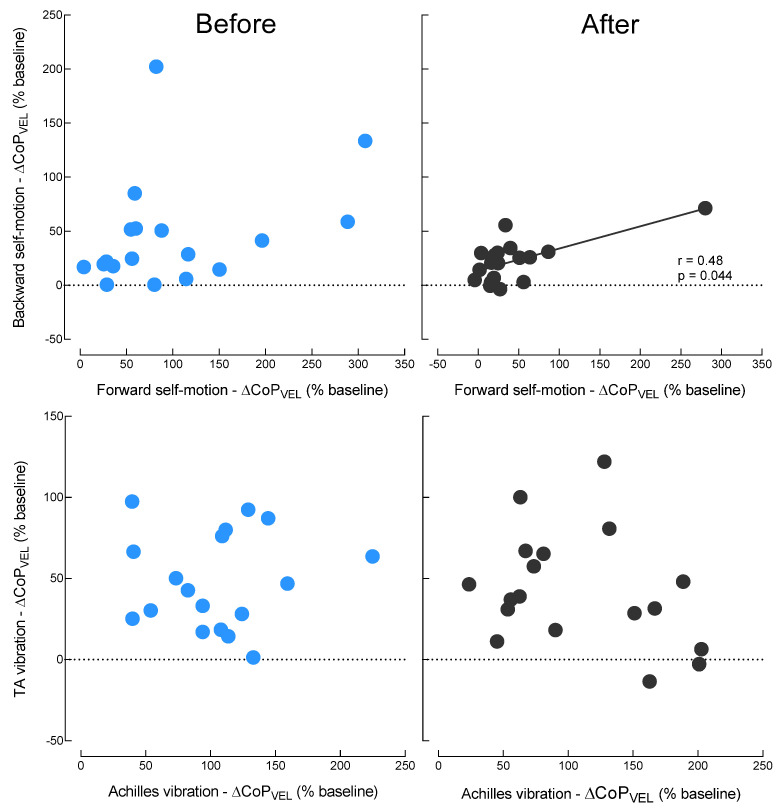
**Intra-modal coherence before and after the intervention.** Scatterplots of changes in center of pressure velocity (expressed as percentage of baseline; ΔCoP_VEL_), between simulated backward and forward self-motion ((**top**) panels) and between response to tibialis anterior (TA) and Achilles tendon vibration ((**bottom**) panels) before ((**left**) column) and after ((**right**) column) the six bouts of optic flow in virtual reality. The regression line shows a significant correlation. Data points represent individual participants.

Before the intervention, no correlation was observed (r = 0.38; *p* = 0.117) between ΔCoP_VEL_ induced by simulated forward and backward self-motion (Figure 5, bottom left panel). Similarly, no correlation was observed (r = 0.00; *p* = 1.000) between ΔCoP_VEL_ induced by TA and Achilles tendon vibration before the intervention (Figure 5, top left panel). After the intervention, the ΔCoP_VEL_ induced by simulated forward and backward self-motion was correlated (r = 0.48; *p* = 0.044). In contrast, the ΔCoP_VEL_ induced by Achilles and TA tendon vibration remained uncorrelated after the intervention (r = −0.27; *p* = 0.284) (Figure 5, right panels).

**Figure 6:**
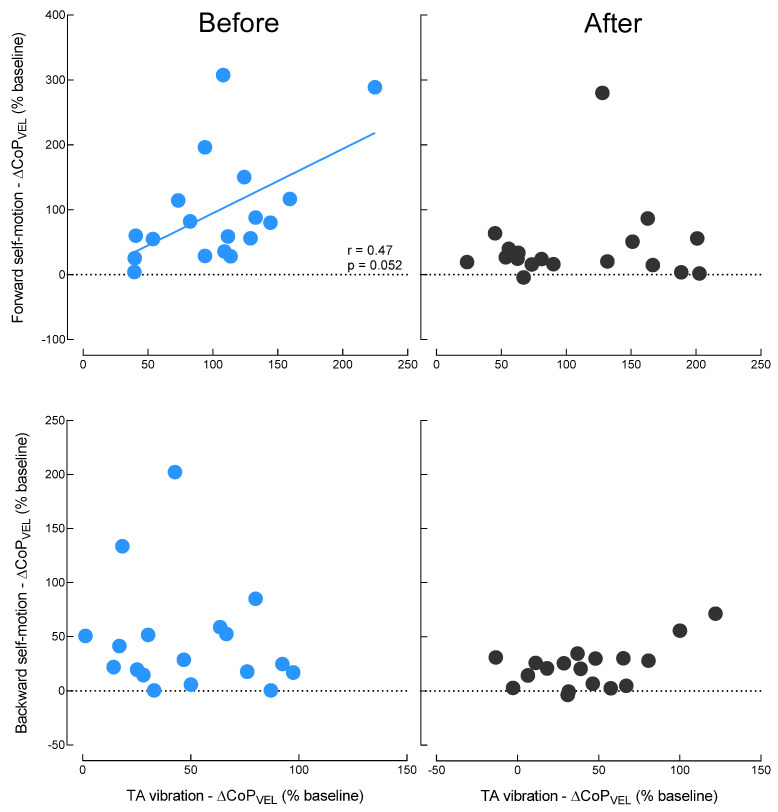
**Inter-modal coherence before and after the intervention.** Scatterplots of changes in center of pressure velocity (expressed as percentage of baseline; ΔCoP_VEL_), between response to simulated forward self-motion and Achilles tendon vibration ((**top**) panels) and between response to simulated backward self-motion and TA tendon vibration ((**bottom**) panels) before ((**left**) column) and after ((**right**) column) the six bouts of optic flow in virtual reality. The regression line shows a significant correlation. Data points represent individual participants.

When looking at the correlation between ΔCoP_VEL_ in response to Achilles tendon vibration and simulated forward self-motion before the intervention, a significant correlation was observed (r = 0.47; *p* = 0.052) (Figure 6, top left panel). In contrast, no correlation was observed between simulated backward self-motion and TA tendon vibration (r = −0.17; *p* = 0.505) (Figure 6, bottom left panel). After the intervention, ΔCoP_VEL_ response to Achilles tendon vibration and simulated forward self-motion was uncorrelated (r = −0.16; *p* = 0.526) as the response to TA tendon vibration and simulated backward self-motion (r = 0.32; *p* = 0.203) (Figure 6, right panels).

## 4. Discussion

The present study demonstrates that an acute intervention consisting of six bouts of optic flow exposure in virtual reality induces measurable adaptations of postural control in healthy older adults. Specifically, repeated exposure to optic flow reduced the postural response to visually simulated self-motion (forward and backward) as indexed by decreases in ΔCoP_VEL_. In addition, postural stability improved when standing in an eyes-closed but not eyes-open stance. Finally, the intervention altered the sensory strategy with the emergence of an intra-modal relation for vision (forward vs. backward optic flow responses became correlated), and the withdrawn of the inter-modal relation involving backward sway. These findings suggest that sensory reweighting retains plasticity in older adults when repeatedly challenged by a visually provocative environment, possibly moving older adults toward a sensory-strategy profile closer to that reported in young adults.

### 4.1. Reduced Postural Response to Optic Flow

In virtual reality balance research, Assländer and colleagues emphasized that optic flow provides a practical approach to estimate the visual contribution to standing balance, thereby offering a means to study the dynamics of the sensory reweighting process [2]. A central result of the present study is the consistent reduction in CoP velocity responses to both forward and backward simulated self-motion after the intervention, accompanied by reductions in CoP excursion. This suggests that repeated exposure to optic flow downregulated the gain linking visual motion cues to compensatory postural adjustments, which is consistent with the idea that the CNS scales the contribution of a sensory channel according to its contextual reliability [1,29]. Importantly, the present findings converge with prior evidence that postural instability is elicited by immersive virtual reality. Fransson and colleagues reported reduced postural instability across repeated exposures to a provocative “rollercoaster”, supporting the concept of visual desensitization and improved handling of sensory conflict [6]. In a closely related protocol, Barbanchon et al. showed that six 90-s bouts of simulated forward/backward self-motion decreased CoP velocity responses to optic flow in young adults [7]. The current study extends this exposure-induced attenuation of optic flow responses to older adults, a group often characterized by increased visual dependence for balance [9,16].

However, the question remains whether the reduction in optic flow responses reflects habituation or sensory reweighting. Converging evidence points toward a contribution of sensory reweighting mechanisms. Theoretical frameworks and computational models [1,29], together with empirical findings [3,4,5,30,31], indicate that inputs from distinct sensory channels are integrated, with their relative contribution being dynamically adjusted according to the functional reliability of each channel. Under such conditions, repeated exposure to a comparable postural disturbance induced by optic flow is expected to reduce the weighting of less reliable inputs (i.e., visual information) with a concomitant increase in the weighting of more reliable sources (i.e., proprioceptive and vestibular information). In contrast, the afferent signals originating from proprioceptive mechanoreceptors are likely interpreted as particularly reliable when standing on a rigid and stable support surface (force platform). It is well established that both muscle afferents [8,22,31] and cutaneous receptors [32,33,34] are critical for postural regulation. The high fidelity of these signals probably promotes a transition from a self-motion percept to a perception of visual motion, consistent with a sensory reweighting process characterized by a down-weighting of visual cues [35]. Finally, the observed reduction in CoP velocity during quiet standing with eyes closed following repeated VR exposure (see below) cannot be accounted for solely by habituation (see below). In agreement, Ahuja et al. showed that repeated sinusoidal optic flow perturbations can reshape postural responses over repeated exposures without evidence of habituation [36].

### 4.2. Decreased Postural Instability in Eyes-Closed Standing

A second key finding is the selective reduction in CoP velocity during quiet standing with eyes closed after the intervention, whereas no change was observed in the eyes-open condition or Romberg ratio. This selectivity argues against a nonspecific “global improvement” and instead suggests a shift toward more effective use of non-visual cues when vision is unavailable. This is consistent with the framework of a dynamic multisensory postural control, which processes and reweights sensory inputs depending on their reliability [1]. In parallel, gastrocnemius medialis EMG decreased in eyes closed (but not eyes open), whereas soleus and tibialis anterior EMG did not change significantly, suggesting a reduction in the neuromuscular “cost” of stabilization under visual deprivation. This supports the interpretation that repeated optic flow exposure can have an impact beyond the virtual environment and benefit non-visual stance control in a real environment in older adults, as observed previously [7]. However, whether the present results indicate a decreased reliance on visual inputs during upright standing remains an open question. Finally, the effect of the repeated exposure to optic flow on balance control mainly emerged from CoP velocity rather than amplitude, supporting previous work highlighting the greater predictive value of CoP velocity for balance capacity [11,37,38]. Overall, an improvement specifically in eyes-closed standing is consistent with the idea that repeated exposure to unreliable visual motion cues can promote a more effective balance strategy in old adults.

### 4.3. Potential Changes in Sensory Strategy

Beyond reduced response magnitudes, the result raises the possibility of reorganization of the sensory strategy. After the intervention, responses to forward and backward optic flow became significantly correlated, whereas they were not correlated before the intervention. This indicates an increased internal consistency within the visual modality after repeated exposure, in agreement with the idea that intra-modal relations reflect modality-specific processing stability based on dedicated internal models [1]. In a previous work, we reported that intra-modal reweighting existed in young but not older adults [13]. Therefore, the repeated exposure to optic flow in older adults could be a means to restore the existing postural strategy exhibited by young adults. However, this interpretation should be considered with caution, as the correlation found after the intervention was mainly due to one participant, which stretched the data dispersion. When this participant was removed from the analysis, no correlation emerged, suggesting that the effect may exist but could be quite modest. Alternatively, intra-modal consistency between postural responses to forward and backward optic flow may reflect a reduced dimensionality of control, whereby behavior is governed by a single visuo-postural gain rather than by direction-specific tuning. This fits with the view that effective postural regulation depends on preserving multiple coordinative solutions rather than collapsing into one invariant mapping. Nonetheless, the fact that intra-modal correlations were observed in young healthy adults for both visual and proprioceptive perturbations supports a functional rather than dysfunctional role of such intra-modal coherence [13]. Further work is, however, necessary to fully understand the meaning of intra-modal coherence in postural control. Interestingly, intra-modal coherence only appeared for the visual perturbation (simulated forward/backward self-motion), suggesting that the change in sensory strategy may be specific to the intervention.

Critically, this new intra-modal structure was accompanied by the disappearance of an inter-modal correlation. Before the intervention, older adults exhibited a significant positive correlation between Achilles tendon vibration and simulated forward self-motion, a pairing that shares a common directional effect on postural output (both primarily induce backward CoP shift). The absence of such coupling in young adults [13] can be interpreted as offering greater neural flexibility for maintaining balance in complex environments, which appears to be reduced in older adults. This suggests that repeated optic flow exposure may reduce a stereotyped cross-modal “shared gain” phenotype and promote more modality-specific organization of balance responses. However, it may also indicate reduced multisensory integration and diminished context-dependent reweighting, given that effective postural control normally depends on flexible coordination across sensory sources and task constraints rather than on fragmented sensory mapping. Although the present study does not allow us to distinguish between these hypotheses, the absence of inter-modal coherence in young adults [13] raises the possibility that the natural configuration, in our experimental conditions, is one without inter-modal correlation; this interpretation, however, requires further investigation. The persistent weakness of backward-related inter-modal relations may reflect direction-specific factors. As mentioned above, backward self-motion cues are generally less ecologically familiar than forward displacement [14], and fear of falling backward may alter postural control dynamics [15,39].

### 4.4. Methodological Considerations and Perspectives

Several methodological aspects should be considered. First, participants were healthy older adults without recent falls and under strict exclusion criteria, which limits generalization to fall-prone populations and clinical groups in whom sensory reweighting profiles may differ. For example, altered sensory reweighting has been documented in fall-prone older adults, with Craig and Doumas reporting larger and longer postural aftereffects and delayed perception of platform stabilization in fall-prone elders [40], while van den Hoorn et al. showed more random CoP dynamics in those prone to falling after vibration offset when dynamic sensory reweighting is required [20]. Second, vection was not directly assessed, which prevents us from determining whether the observed postural adaptation reflected changes in perceived self-motion, especially given that vection and postural responses can vary independently under optic flow stimulation [41] and may rely on partly distinct mechanisms [42]. Finally, while CoP velocity is a reliable primary outcome [38] and clinically meaningful [37], complementary analyses (e.g., longer trials allowing additional decomposition approaches) could refine mechanistic inference.

A further limitation is that several a priori defined outcomes were examined, and although these outcomes captured distinct aspects of postural control, the number of statistical comparisons performed across outcomes may have increased the risk of type I error at the study level. Accordingly, the findings, particularly those based on secondary outcomes or correlation analyses, should be interpreted with caution and confirmed in future studies with larger samples. In addition, considering the quite small sample size, the present results should be considered cautiously, although the large size effect reported supports genuine changes after the intervention.

Future studies should test dose–response and retention to determine the effect of the number of bouts on the magnitude and the duration of the observed balance improvements in eyes-closed stability. Furthermore, combining optic flow exposure with manipulations of proprioceptive reliability or vestibular challenge would help determine whether the eyes-closed benefits reflect true up-weighting of non-visual cues or broader changes in control gains and corrective strategies.

## 5. Conclusions

In summary, six bouts of optic flow exposure in virtual reality reduced visually-induced postural destabilization and improved quiet-standing stability when vision was removed in healthy older adults. In addition, the results suggest a reorganization of sensory strategy structure with the emergence of visual intra-modal consistency after the intervention, while a pre-existing inter-modal coupling between Achilles vibration and forward optic flow disappeared. These findings reinforce the view that sensory reweighting and strategy organization remain modifiable in older age with a VR environment. These findings open promising avenues for future research into its application in rehabilitation settings, with potential implications for optimizing intervention protocols aimed at improving or restoring balance capacity in healthy and clinical populations.

## Figures and Tables

**Table 1:** **Center of pressure amplitude during upright standing before and after the intervention.** Center of pressure amplitude in antero-posterior (AP) and medio-lateral directions (ML), and corresponding mean rectified EMG activity for the soleus (SOL), gastrocnemius medialis (GM), and tibialis anterior (TA) before and after the 6 bouts of optic flow in virtual reality when standing with eyes open and eyes closed.

	Eyes Open				Eyes Closed			
	Before		After		Before		After	
AP (mm)	8.0	(5.6)	7.8	(4.5)	8.5	(9.1)	10.5	(6.7)
ML (mm)	6.0	(3.0)	5.5	(4.1)	6.1	(3.6)	7.0	(3.9)
SOL (% MVC)	18.4	(7.7)	18.8	(12.5)	18.7	(11.5)	17.5	(11.0)
GM (% MVC)	12.0	(10.1)	11.3	(8.8)	13.4	(12.0)	12.1	(11.8) *
TA (% MVC)	2.7	(2.5)	2.6	(1.9)	2.8	(1.4)	2.2	(1.9)

* denotes statistical difference with before (*p* < 0.05). Data are expressed as median (interquartile range).

**Table 2:** **Effect of simulated self-motion on maximal amplitude of the center of pressure before and after the intervention.** Maximal amplitude of the center of pressure in antero-posterior (AP) and medio-lateral (ML) during simulated forward and backward self-motion, and Achilles and tibialis anterior (TA) tendon vibration before and after the 6 bouts of optic flow in virtual reality.

	Simulated Forward Self-Motion				Simulated Backward Self-Motion			
	Before		After		Before		After	
AP (mm)	18.2	(9.1)	11.5	(5.6) *	12.9	(8.7)	12.1	(9.1) *
ML (mm)	11.2	(5.8)	8.2	(3.5) *	6.9	(4.0)	6.4	(3.1)
		**Achilles tendon vibration**				**TA tendon vibration**		
AP (mm)	29.2	(13.1)	28.9	(13.5)	23.2	(10.2)	24.5	(14.1)
ML (mm)	12.2	(6.3)	13.2	(5.9)	8.9	(6.6)	12.1	(7.7)

* denotes statistical difference with before (*p* < 0.05). Data are expressed as median (interquartile range).

## Data Availability

The datasets presented in this article are not readily available. Requests to access the datasets should be directed to stephane.baudry@ulb.be.

## Abbreviations

AP	Antero-posterior
CoP	Center of pressure
CoPamp	Maximal amplitude of center of pressure displacement
CoPvel	Mean center of pressure velocity
EC	Eyes closed
EMG	Electromyography/electromyogram (surface EMG recordings)
EO	Eyes open
GM	Gastrocnemius medialis
Ia	Group Ia muscle spindle afferents
ML	Medio-lateral
MVC	Maximal voluntary contraction
SOL	Soleus muscle
TA	Tibialis anterior
VR	Virtual reality
